# The radiologic diagnosis of skeletal dysplasias: past, present and future

**DOI:** 10.1007/s00247-019-04533-y

**Published:** 2020-11-02

**Authors:** Amaka C. Offiah, Christine M. Hall

**Affiliations:** 1 Academic Unit of Child Health, Department of Oncology & Metabolism, University of Sheffield, Western Bank, Sheffield, S10 2TH UK; 2 Institute of Child Health, University College London, London, UK

**Keywords:** Child, Database, Medical history, Ontology, Radiology, Skeletal dysplasia

## Abstract

Skeletal dysplasias have been recognised since recorded history began. The advent of radiography at the beginning of the 20th century and the subsequent introduction of departments of radiology have had tremendous impact and allowed conditions to be identified by their specific radiographic phenotypes. This has been enhanced by the addition of cross-sectional modalities (ultrasound, computed tomography and magnetic resonance imaging), which have allowed for prenatal recognition and diagnosis of skeletal dysplasias, and by the recent explosion in identified genes. There are more than 400 recognised skeletal dysplasias, many of which (due to their rarity) the practising clinician (radiologist, paediatrician, geneticist) may never come across. This article provides a historical overview of aids to the radiologic diagnosis of skeletal dysplasias.

## The miraculous impact of Röntgen rays

**Fig. 1:**
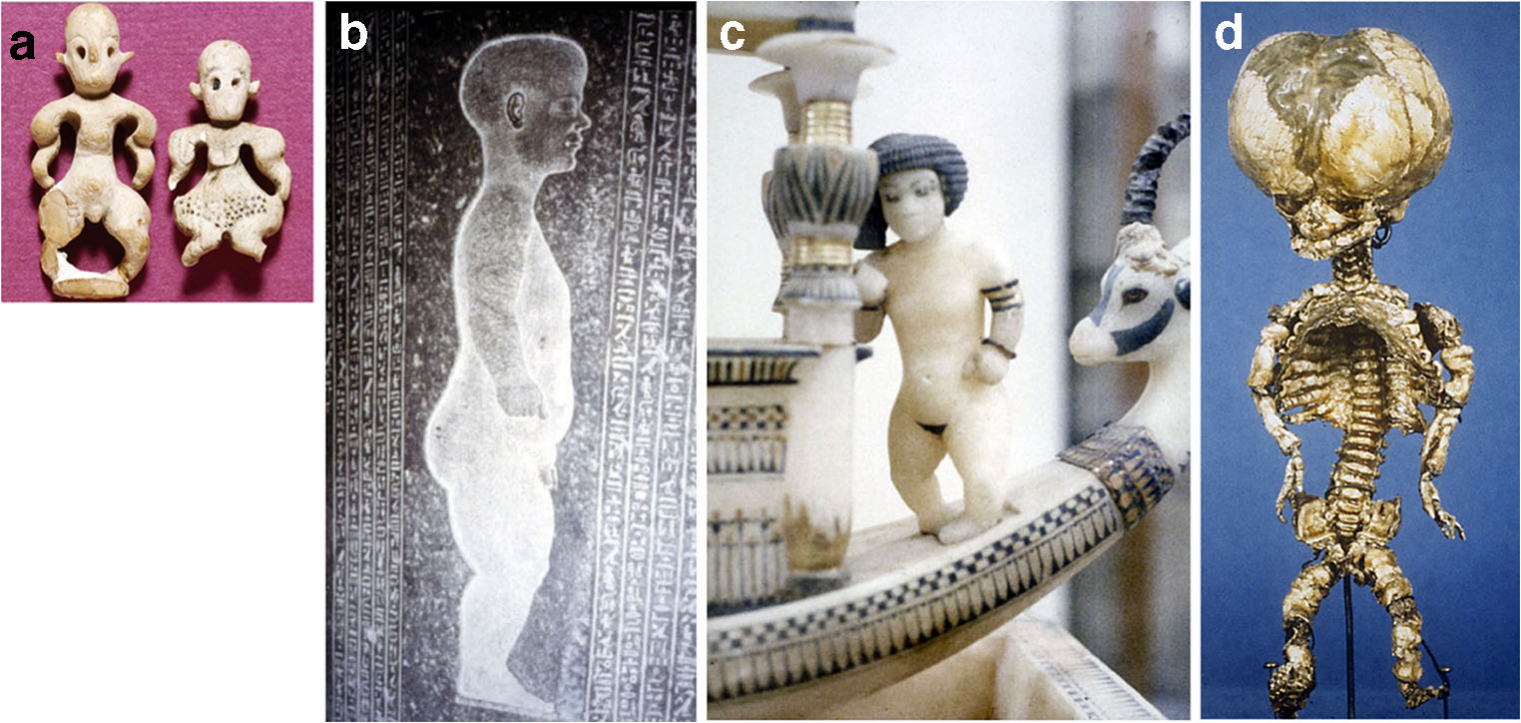
Skeletal dysplasias through the ages. **a** Figures of male (*left*) and female (*right*) dwarfs from Egypt were made from hippopotamus ivory from the Predynastic Naqada II Period (3500–3200 BCE). **b** Granite stela of the dwarf Djheo from the Late Period (750–332 BCE). Short stature was not regarded as a physical deformity but as a divine mark, therefore dwarfs wanted their likeness to be depicted on their stele. Djheo (a native Egyptian) is characterised as having achondroplasia. **c** Carved from alabaster, this is a typical dwarf of Amarna (capital of Akenten) from the tomb of Tutenkhamun, exhibited in the Cairo Museum. The severe talipes and flexed arms suggest diastrophic dysplasia. **d** A historical specimen of a fetus with osteogenesis imperfecta type 2, exhibited in the Vienna Pathology Museum

Skeletal dysplasias have been recognised since recorded history began. There are carved ivory statuettes of individuals with achondroplasia as early as the Predynastic Period in ancient Egypt from more than 6 millennia ago. In the Early Dynastic Period (about 3,000 BCE) the statues and carvings were true representations of the human form and achondroplasia was clearly recognisable (Fig. 1). Since then, people of short stature have played important roles in society, including as portents of good luck, workers in precious metals, servants in royal households, jesters, jugglers and actors. More recently, classification of skeletal dysplasias was begun by the great anatomists and pathologists of the 17th to 19th centuries, although many dysplasias were mistakenly diagnosed as rickets or syphilis. The larger displays are in the Rondemuseum (a former mental asylum) in Vienna, the Berlin Museum of Medical History (housing the Virchow Collection) and the Museum Vrolik in Amsterdam.

“I have seen my death!” exclaimed Anna Röntgen in 1895 when her husband showed her a radiograph of her hand; it must have seemed a miracle to her. In fact, the advent of radiography at the beginning of the 20th century and the subsequent introduction of departments of radiology, have had tremendous (if not miraculous) impact and allowed conditions to be identified by their specific radiographic phenotypes. Until this time, conditions had mainly been defined by their clinical phenotypes, leading to many different conditions being named as achondroplasia, including Morquio disease and spondyloepiphyseal dysplasia congenita. Radiographs allowed these conditions to be disentangled. For example, in 1959, Maroteaux and Lamy [1] published the first description of pseudoachondroplasia as a distinct radiologic phenotype.

The explosion in identification of distinct conditions that came with the discovery of Röntgen rays was added to by the advent of cross-sectional modalities (ultrasound [US], computed tomography [CT] and magnetic resonance imaging [MRI]), which have allowed the prenatal recognition and diagnosis of skeletal dysplasias [2]. Along with this came developments in genetics, with a matched (or possibly exceeded) expansion in identified genes.

The structure of DNA was identified in 1953 and during the 1950s, specific patterns of the nucleotides, represented by four letters (A, T, G and C), were described. A and T always appear in equal measures, as do G and C, and this led to the description of the double helix shape of DNA [3]. Following the discovery of chromosomal changes in the early 1960s [4], medical genetics experienced rapid expansion.

The Human Genome Project, a 13-year international collaboration, resulted in the complete sequencing of the human genome in 2001 [5]. This identified that humans have about 23,000 protein-coding genes, which is only 1.5% of the entire genome. The rest is made up of what has been called “junk” DNA. Now we realise that more than 80% of the genome is biologically active, with much non-protein-coding DNA regulating nearby genes. The genetic basis of many diseases may not be in protein-coding genes at all but in their regulatory neighbours.

**Fig. 2:**
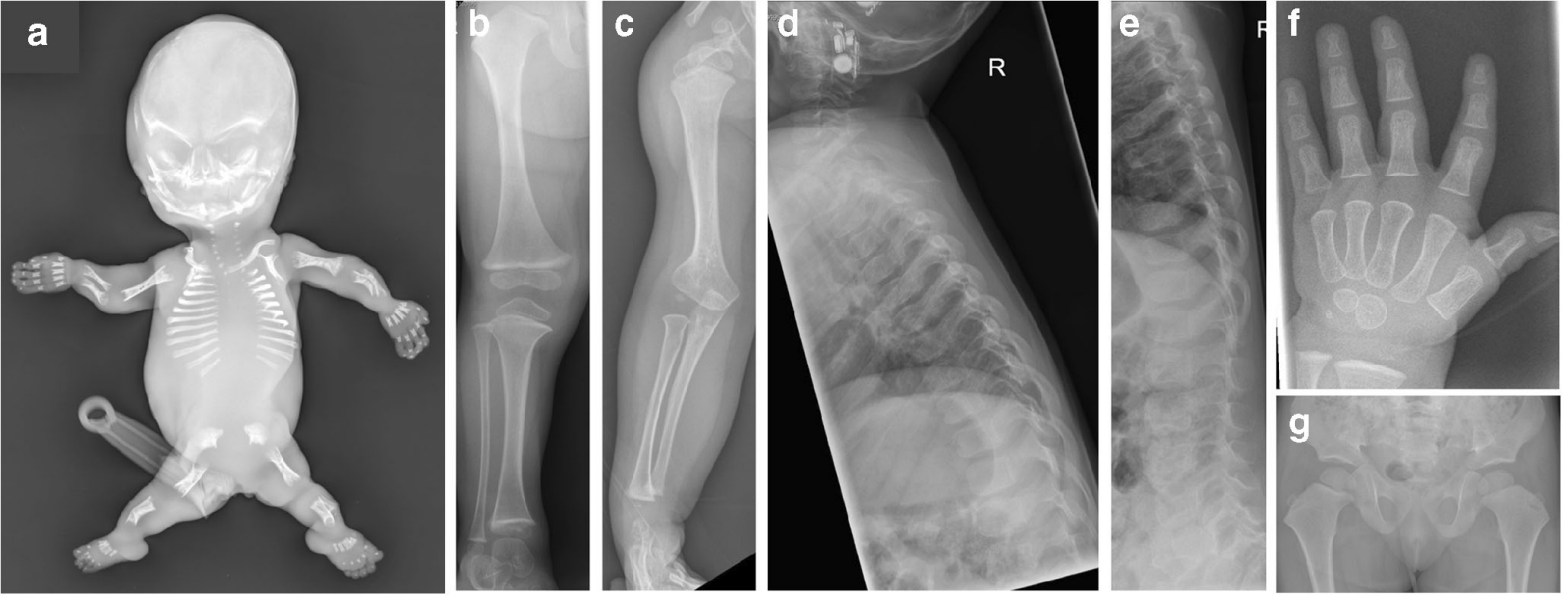
The range of Type 2 collagen disorders spans from lethal achondrogenesis type 2 (**a**) to the relatively mild Stickler syndrome (**b-g**), in which final height may be within normal limits. **a** Anteroposterior (AP) radiograph of a 17 gestational-week fetus shows typical features of achondrogenesis type 2. **b-g** 3-year-old boy with Stickler syndrome. AP radiograph of the right lower extremity (**b**) shows wide metaphyses of the lower lmb. AP radiograph of the right upper extremity (**c**) shows wide metaphyses of proximal and distal humerus. AP (**d**) and lateral (**e**) radiographs of the spine show mild narrowing of intervertebral disc spaces. Note the absence of platyspondyly. Posteroanterior radiograph of the left hand (**f**) shows wide metaphyses of the metacarpals. Delayed ossification of the epiphyses of metacarpals and phalanges. AP radiograph of the pelvis (**g**) shows broad femoral necks

During the 1980s, there was the wide recognition of families of disorders. These families have certain characteristics in common and are the result of different mutations in the same gene. One example is the Type 2 collagen family ranging from lethal achondrogenesis Type 2 through spondyloepiphyseal dysplasia congenita to the milder Stickler syndrome (Fig. 2). While one gene may result in many phenotypes, it has also become clear that one clinical phenotype, for example osteogenesis imperfecta, although usually the result of mutations in Type 1A collagen, may be caused by as many as 33 different gene mutations [6].

There are more than 400 recognised skeletal dysplasias [7], many of which (due to their rarity) the practising clinician (radiologist, paediatrician, geneticist) may never come across, and this is true even for clinicians with a subspecialist interest in the field.

Many dysplasias are unknown and/or unique to a single family and have no specific nomenclature. The situation is further complicated by the fact that some conditions evolve with age and therefore important radiographic clues present in the neonate may be absent in the younger child, making the diagnosis more difficult. For example, the Weissenbacher-Zweymüller neonatal phenotype evolves to a normal radiologic phenotype in infants and young children and then further evolves to either Stickler syndrome or otospondylomegaepiphyseal dysplasia as the child ages [8]. The converse is also true; for example, increasingly striking radiographic changes are seen in metaphyseal chondrodysplasia type Jansen as the child gets older [9]. Finally, many skeletal dysplasias are difficult to diagnose after physeal closure, with changes of secondary osteoarthritis (for example) being the only radiographic feature.

Given all of the above, a paediatric radiologist faced with the abnormal skeletal survey of an individual with a skeletal dysplasia – all of which are “rare” (defined as having a prevalence of less than 1 in 2,000 people) -- may not have come across the condition previously and yet may be asked to attempt a diagnosis, if only to direct the precise genetic mutation(s) to exclude or search for. If the radiologist is uncertain, they will want to access support. This article provides a historical overview of aids to the radiologic diagnosis of skeletal dysplasias.

## The magic and authority of words

Whether one-on-one or in small or large groups, the verbal exchange of knowledge from teacher to pupil or between experts will always be an important resource. Those wishing to develop expertise in the field of skeletal dysplasias will benefit from shadowing recognised experts and are strongly encouraged to attend relevant meetings and conferences. These provide an important opportunity to revise existing knowledge, to catch up with the latest developments, to present difficult/unknown cases to colleagues who may help to make the diagnosis and finally they provide an opportunity to interact with others with similar interests, establishing strong links and collaborations.

In 1979, the Skeletal Dysplasia Group for Teaching and Research (SDG) was founded in the United Kingdom by the amalgamation of the Metabolic Bone Group of Great Ormond Street Hospital for Sick Children and the Skeletal Dysplasia Group of the British Paediatric Orthopaedic Society [10]. The International Bone Dysplasia Society was founded in Bad-Honeff in 1991 and formalised in 1993 in Chicago. Since then, biennial meetings have been held, either in the United States or in Europe. In 1999, at the meeting in Baden-Baden, the International Skeletal Dysplasia Society (ISDS) was founded. This society includes geneticists, both clinical and molecular, paediatric radiologists, paediatricians and a few endocrinologists, pathologists and orthopaedic surgeons [11]. This representation reflects the wide clinical spectrum needed in the diagnosis and management of patients with skeletal dysplasias. Also, inevitably, a wide skills mix is needed by each diagnostician in this field. For example, a paediatric radiologist in the field of skeletal dysplasias not only has a mastery of skeletal pattern recognition, variations from normal and application to diagnosis, but also some understanding of molecular genetics, matching skeletal features with specific genetic mutations and gene pathways. Specific multispecialty courses for the diagnosis of skeletal dysplasias include those run by the Skeletal Dysplasia Group for Teaching and Research (held in Sheffield, United Kingdom) [10] and the ISDS teaching course in Lausanne, Switzerland [12].

## Paradise is a kind of library

Printed journal articles in the form of case and series reports and review articles are an important resource, but they are limited by the number of conditions they can cover. On the other hand, digital access to these articles (described below) has vastly increased our ability to search specific terms related to phenotype and/or genotype and therefore has increased the importance to clinicians and researchers of published single case and series reports in the field of rare conditions.

One important journal publication (the Nomenclature) deserves specific mention. At the 1970 European Society of Paediatric Radiology meeting in Paris, under the presidentship of Jacques Sauvegrain, a group of interested paediatric radiologists (including John Sutcliffe, Andres Giedion and Kazimierz Kozlowski) met with Juergen Spranger and Pierre Maroteaux to attempt an initial formal classification or nomenclature of Constitutional Disorders of Bone. In 1971, McKusick and Scott [13] in the United States, published a further nomenclature. The nomenclature meetings aimed to bring together a balanced representation of experts in radiology, clinical genetics and paediatrics to agree on the denomination and classification of the skeletal disorders, syndromes and metabolic diseases that were being described at a rapid pace. Much has changed since the first Nomenclature was published in 1970, largely as a result of the identification of molecular changes during the 1970s and ‘80s. Revisions have been prepared in 1977, 1983, 1992, 1997 and every 4 years thereafter, with the most recent publication being in 2019, after the 2017 meeting held in Bruges, Belgium [7]. The number of recognized genetic disorders with significant skeletal changes is even now still increasing and the distinction between dysplasias, metabolic bone disorders, dysostoses and malformation syndromes has become less distinct. In the more recent classifications, pathogenetic and molecular criteria are integrating with morphological changes, but disorders are still identified by clinical and radiographic features.

In 2001, the Nomenclature became the Nosology. The former refers to a name or designation, whereas the latter refers to a classification of disease. This change in terminology reflected the fact that the focus had shifted from merely labelling the dysplasias to classifying them. The Nosology should coexist with other classifications based on the clinical and radiographic approach to diagnosis or based on the molecular changes and pathways, and it is expected that electronic means will facilitate transition and interactions between the various classification criteria.

Textbooks provide high-quality and more exhaustive compilations of skeletal dysplasias than both (personal) notes or handouts from meetings and journal articles. An early expert in clinical diagnosis, delineation and classification of skeletal dysplasias was Sir Thomas Fairbank, an orthopaedic surgeon known in the United Kingdom as the father of skeletal dysplasias, who in 1951 published “An Atlas of General Affections of the Skeleton” [14]. His reviewer wrote, “Sir Thomas Fairbank knows far more about bone disease than anyone else in the country. This is not only because of his many years on the staff of an undergraduate teaching hospital and of the Hospital for Sick Children, Great Ormond Street, but also because he is our orthopaedic father, whose interests we know and to whom we take all our problems and prizes” [15].

In 1964, the radiologist Philip Rubin published his “Dynamic classification of bone dysplasias” [16]. Later, great diagnosticians Pierre Maroteaux (Paris) and Juergen Spranger (Mainz) together with Langer and Wiedemann, and Taybi and Lachman (United States), all paediatricians or paediatric radiologists, independently published textbooks on the skeletal dysplasias in 1974-'75 [17–19]. Later publications ensued, for example in 1985, Apley, Wynne-Davies and Hall (United Kingdom) published their text “Atlas of Skeletal Dysplasias” [20]. While these cover the general topic of skeletal dysplasias, other texts have concentrated on specific aspects, for example Poznanski’s “The Hand in Radiologic Diagnosis” [21], Beighton and Cremin’s book on sclerosing bone dysplasias [22] and Hall et al.’s atlas of fetal skeletal dysplasias [23].

Although textbooks are an extremely useful aid, they suffer from several setbacks. Firstly, developments in the field occur at such a rapid pace that the information is often out of date by the time the books are in print. Secondly, unless they have a gamut’s section, they are not always helpful in “triangulation,” i.e. providing a differential diagnosis based on a combination of specific features. The most important limitation of textbooks, however, is that they cannot exceed a certain size (because of cost and portability). This means authors are limited by the number of conditions and/or number of images they can illustrate. This limitation is now overcome by the development of digital resources that may accompany conventional textbooks or may be standalone, as discussed in the next section.

## Analogue creatures in a digital world

The internet has caused an explosion in digital technology, such that we now have at our fingertips portable technology that allows rapid and widespread knowledge transfer. The digital era has changed the way we live our lives and the way in which we educate ourselves. A significant advantage of digital technology is the ability to rapidly communicate ideas through email. Mailing lists can be created so that everyone with an interest can contribute to the discussion. SkelDys is such a forum, used by the members of the ISDS; it functions as an online forum, through which members communicate by email. Difficult or interesting cases are posted and, by responding to the emails, individual members are able to comment, suggest diagnoses or genes that should be tested, attach relevant publications, link researchers, etc.

In addition to the transfer of ideas, digital technology allows the transfer of images (photographs, radiographs, slides, etc.) via various means including compact discs, digital versatile discs, email and cloud-based systems such as Google Drive and Dropbox. Issues related to consent and data protection, particularly in light of the 2018 European General Data Protection Regulation (GDPR) are outside the scope of this article but should always be considered before the transfer of patient details and images.

Although usually transferred by email in joint photographic experts’ group (JPG/JPEG) or tagged image file (TIFF) formats, if radiographic images are saved/transferred in their original digital imaging and communications in medicine (DICOM) standard format, the recipient can easily download and install DICOM viewer software on their own computer. It is also possible to transfer images via secure networks such as national image exchange portals.

This ready transfer of images, coupled with teleconferencing and videoconferencing facilities, improves access to the limited numbers of radiology experts in the field of skeletal dysplasias and allows virtual (clinical and/or research) meetings to take place in a timely manner at less cost and inconvenience.

Digital technology also allows compilation of cases into teaching files or digital atlases. One such atlas consists of images of 13 skeletal dysplasias and three comparative normal skeletons [24]. Such a small library of conditions may be useful for beginners, but there is no reason for digital resources to be this restricted. As previously mentioned, digital media can overcome the disadvantages of size (and cost) related to textbooks. This includes compact and digital versatile discs, either accompanying textbooks or as standalone resources (for example, the London Dysmorphology Database [25], OSSUM [an illustrated database of skeletal dysplasias] [26] and REAMS, a Radiological Electronic Atlas of Malformation Syndromes and Skeletal Dysplasias [27]). The latter incorporated the temporal reasoning framework for the first time, allowing the user to consider age-specific radiologic findings, an important factor in skeletal dysplasias [28]. These external disc-based databases hold more information and permit more rapid and complex searches than printed textbooks. For example, searches can be performed using terms related to the patient’s clinical phenotype (e.g., sparse hair, rounded nose), radiologic phenotype (cone-shaped epiphyses, brachydactyly), a specific condition (trichorhinophalangeal syndrome Type 1), a specific gene mutation (*TRPS1*) or any combination of these. However, their content remains finite and, like textbooks, compact and digital versatile discs take some time to update and, as a result, cannot keep up with the rapid developments that occur in this field. The same cannot be said for internet-based resources, which can, if necessary, be updated on a daily basis.

The internet is a powerful aid to the diagnosis of skeletal dysplasias, made even more so by bespoke online databases such as the Online Mendelian Inheritance in Man (OMIM), which catalogues human genes and genetic disorders [29], the Rare Disease Database collated by the National Organization for Rare Diseases [30], the London Medical Databases, accessed via Face2Gene as an extension of the previous CD-based London Dysmorphology Database [31], and POSSUM (pictures of standard syndromes and undiagnosed malformations) [32]. These are all excellent databases but vary in the quantity of radiologic images they present.

Most, if not all, clinicians will at some time have used a database, but early on it was shown that they did not necessarily provide any advantage over textbooks and were slower and harder to use [33]. However, the same authors suggested that these parameters would improve with increased user familiarity with databases. This has been shown to be the case, but there remains a limitation.

Correctly identifying a radiologic abnormality may not necessarily lead to a correct diagnosis if the term that is searched for is not the same term used in the database to define that abnormality (for example, if “irregular” is used rather than “fragmented” when describing the capital femoral epiphyses in a child with a form of epiphyseal dysplasia and “irregular” does not appear in the database).

This realisation led to the development of “ontologies” in the skeletal dysplasia/dysmorphology domain. An ontology organises large data sets into categories/concepts and forms relationships between them. For example, an ontology will link “irregular” and “fragmented” to each other, but also to any anatomical site to which they might apply and any condition in which they are seen. In this way, two users may reach the same diagnosis, even if one searches with the term “irregular” and the other with the term “fragmented.” If the ontology is online, it can readily be updated to include other relevant terms. In the same way, if terms are not linked, then the user will come to learn that “stippled” should not be used when “irregular” or “fragmented” is meant. An ontology becomes even more powerful if these descriptive terms are used to annotate relevant images.

The Human Phenotype Ontology (HPO) is one of (if not) the largest existing medical ontology [34], but it is limited in terms of the radiologic findings seen in skeletal dysplasias. The Bone Dysplasia Ontology aims to integrate genotypic and phenotypic findings in skeletal dysplasias [35], while the dynamic Radiological Electronic Atlas of Malformation Syndromes (dREAMS) [36] provides a detailed radiologic ontology for skeletal dysplasias and is planned to be linked to the Human Phenotype Ontology [34] and used for the UK-based 100,000 Genomes Project [37]. The dynamic Radiological Electronic Atlas of Malformation Syndromes and Skeletal Dysplasias currently consists of more than 15,500 images (mostly radiographs, but some US, CT and MRI images) and approximately 340 conditions. Access to dREAMS is expected to be available in 2020.

Because of the links between concepts that an ontology allows, it can make inferences. This ability differentiates a knowledge base (which stores knowledge) from a database (which stores data) and a knowledge base can be said to be a form of artificial intelligence.

Because artificial intelligence algorithms can be developed to automatically identify complex signal and shape patterns from medical images, analyse vast amounts of data and produce quantitative results, there is huge interest in the applications of artificial intelligence to radiologic tasks. Indeed, authors have recently asked whether it is a threat to radiologists; they conclude that it is not, but that it will change our role, allowing us to take on more value-added tasks [38].

**Fig. 3:**
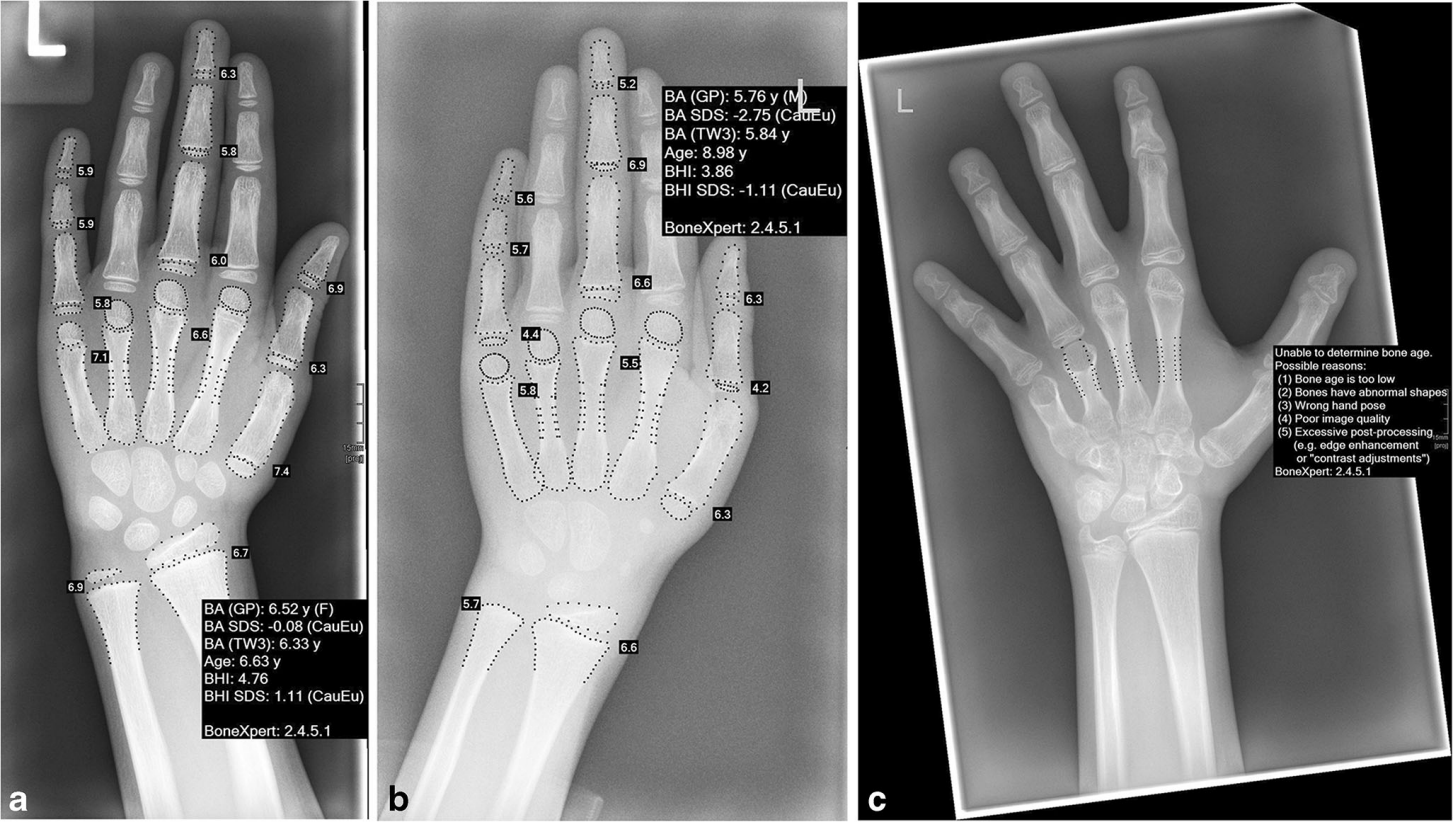
BoneXpert interpretation of left-hand radiographs. **a** Dorsopalmar left hand and wrist in a 6-year, 7-month-old girl with hypophosphatasia. Her bone age is within normal limits (0.08 standard deviations below the mean). **b** Dorsopalmar left hand and wrist in an 8-year, 11-month-old boy with short stature. His bone age is delayed (2.75 standard deviations below the mean). **c** Dorsopalmar left hand and wrist in an 11-year, 8-month-old girl whose radiograph shows short fourth and fifth metacarpals, previous cone-shaped epiphysis of the middle phalanx of the index finger and short terminal phalanges. BoneXpert was not able to determine bone age in this child with dysmorphic bones

A good example of this is the BoneXpert software programme [39]. The software is now in widespread use and seamlessly integrates with hospital picture archiving and communications systems (PACS). It automatically provides the Greulich and Pyle bone age, the bone age standard deviation score, the Tanner and Whitehouse 3 bone age and the bone health index from a hand and wrist radiograph. While BoneXpert rapidly provides the bone age, it does not assess the morphology of the bones and in the presence of a dysplasia is not always able even to provide the bone age (Fig. 3). The radiologist is still required to review the hand and wrist radiograph and correlate any abnormal findings with clinical features and other abnormality on the remainder of the skeletal survey.

Bone Health Index is automated radiogrammetry and may potentially be used to predict fracture risk in children [40], although use of the manual technique suggests otherwise [41].

SpineAnalyzer is a software tool used to detect vertebral fractures from radiographs (or more commonly DXA in adults). Although it has been shown to be unreliable in children [42], it is another example of the potential for artificial intelligence in the diagnosis of skeletal dysplasias. If software can be developed to recognise vertebral fractures, can it potentially be trained to recognise platyspondyly, humped or beaked vertebral bodies and (away from the spine) trident acetabula, chevron epiphyses, cloverleaf skull and more? If so, will there remain a role for the radiologist with an interest in skeletal dysplasias?

## The future’s bright – or is it?

**Fig. 4:**
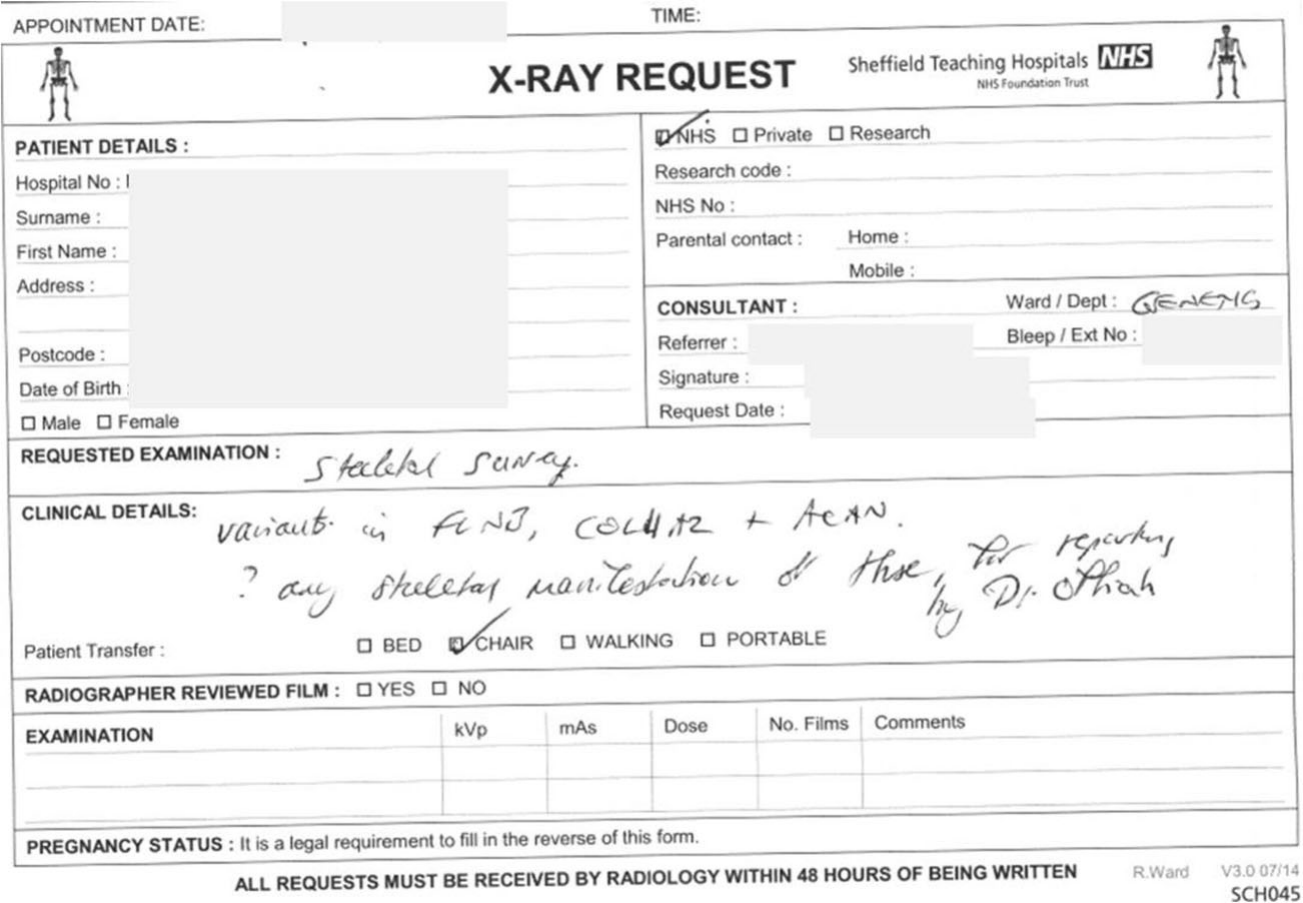
“Reverse radiology” in practise. Mutation analysis identified variants in three genes for which the geneticist required author A.C.O. to review the skeletal survey for phenotypic correlation

Is there a future role for the radiologist in diagnosing skeletal dysplasias or will the combination of artificial intelligence and whole-exome sequencing banish us to history? For many years, molecular genetic confirmation has been the last step in the diagnostic pathway. However, the analysis of gene panels has already replaced single-gene analysis in most instances. Even broader approaches, such as that of whole-exome sequencing and even whole-genome sequencing, are now being used as first-line investigations. For this reason, a wide skills mix is needed by each diagnostician in the field of skeletal dysplasias. For example, a paediatric radiologist not only has a mastery of skeletal pattern recognition, variations from normal, age-dependent morphological changes and application to diagnosis, but also an understanding of molecular genetics matching skeletal features with specific genetic mutations and gene pathways. This cross-specialisation will become more important for diagnosis as we are increasingly being asked to assess the molecular findings and to match them to the radiographic and clinical phenotypes. This approach is now such a common aspect of clinical practice (Fig. 4) that the first author (A.C.O.) uses the term “reverse radiology” to describe it. The practise of reverse radiology requires as much radiologic expertise for correlating clinical and radiographic features with molecular findings as does the more conventional radiology practise of providing diagnostic possibilities for later molecular testing.

The conclusion, therefore, is that just as in the past, there remains a clear and important present and future role for the paediatric radiologist in the diagnosis of skeletal dysplasias.
